# Effect of Tow Size and Interface Interaction on Interfacial Shear Strength Determined by Iosipescu (V-Notch) Testing in Epoxy Resin

**DOI:** 10.3390/ma11091786

**Published:** 2018-09-19

**Authors:** Filip Stojceveski, Andreas Hendlmeier, James D. Randall, Chantelle L. Arnold, Melissa K. Stanfield, Daniel J. Eyckens, Richard Alexander, Luke C. Henderson

**Affiliations:** 1 Institute for Frontier Materials, Deakin University, Waurn Ponds Campus, Geelong 3216, Australia; ajhendlm@deakin.edu.au (A.H.); jdran@deakin.edu.au (J.D.R.); carno@deakin.edu.au (C.L.A.); mstanfie@deakin.edu.au (M.K.S.); deyckens@deakin.edu.au (D.J.E.); 2 Centre for Regional and Rural Futures, Deakin University, Waurn Ponds Campus, Geelong 3216, Australia; richard.alexander@deakin.edu.au

**Keywords:** carbon fiber, interfacial shear strength, interfacial evaluation

## Abstract

Testing methodologies to accurately quantify interfacial shear strength (IFSS) are essential in order to understand fiber-matrix adhesion. While testing methods at a microscale (single filament fragmentation test—SFFT) and macroscale (Short Beam Shear—SBS) are wide spread, each have their own shortcomings. The Iosipescu (V-notch) tow test offers a mesoscale bridge between the microscale and macroscale whilst providing simple, accurate results with minimal time investment. However, the lack of investigations exploring testing variables has limited the application of Iosipescu testing to only a handful of studies. This paper assesses the effect of carbon fiber tow size within the Iosipescu tow test for epoxy resin. Tow sizes of 3, 6, and 9 k are eminently suitable, while more caution must be shown when examining 12, and 15 k tows. In this work, tows at 18 and 24 k demonstrated failure modes not derived from interfacial failure, but poor fiber wetting. A catalogue of common fracture geometries is discussed as a function of performance for the benefit of future researchers. Finally, a comparison of commercial (T300), amine (T300-Amine), and ethyl ester (T300-Ester) surface modified carbon fibers was conducted. The outcomes of this study showed that the Iosipescu tow test is inherently less sensitive in distinguishing between similar IFSS but provides a more ‘real world’ image of the carbon fiber-epoxy interface in a composite material.

## 1. Introduction

**Figure 1:**
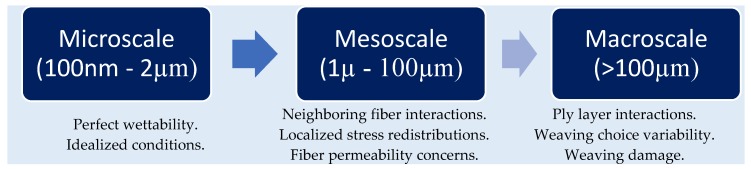
Graphic representing the different scales of testing and introduced testing variabilities.

Composite materials are becoming ever more prominent within engineering applications for their high strength and light weight properties, however limitations to performance still remain. One such limitation is caused by poor fiber-to-matrix adhesion, which limits the ability of stress to be transferred from the matrix to the supporting high strength fibers [1,2,3]. Stresses in a composite can easily surpass that of the interfacial bond strength at the fiber matrix junction thereby leading to premature failure via debonding. In addressing interfacial debonding, a large body of research has focused on modifying both fibers and resin properties to promote interface adhesion and fiber/matrix compatibility [4,5,6,7,8,9,10]. For research in this field to be of importance now and into the future, accurate testing methods to quantify IFSS are required (Figure 1).

The Iosipescu shear test can determine interfacial shear strength (IFSS) of composite materials, and was recently reviewed [11]. Also known as the V-notch shear test, this protocol creates a region of pure shear across a notched sample for which the mechanical properties of a material can be quantified as a measure of specimen geometry and failure load [12,13,14,15]. Although not as commonly implemented as single fiber fragmentation testing (SFFT) or short beam shear testing (SBS), the Iosipescu tow test has several advantages.

Where SFFT provides a perfectly wetted fiber for interfacial evaluation [16], the Iosipescu method introduces tow bundle testing, which investigates neighboring fiber interactions and wetting variability. This is more representative of real-world composites than microscale testing. Additionally, SFFT requires a matrix that has an elongation 3 times larger than that of the embedded fiber. This resin must also be transparent to allow for visualization of the single filament under polarized light. The Iosipescu tow test had the added advantage over SFFT of being applicable to non-transparent resins and allows strict consideration of fiber to fiber interaction.

When considering the SBS, concerns are often raised relating result accuracy. Stress concentrations about the roller supports and mixed-mode bending are both commonly observed to created complex stress distributions that may not provide the most representative conditions of interfacial shear testing [17,18,19]. Iosipescu testing removes these concerns by creating a region of pure shear stress across the center of the specimen. Furthermore, as it is not a time intensive method to manufacture or test V-notch shear specimens, compared to SFFT, and provides design versatility (testing can be conducted as both laminates or embedded tow), the Iosipescu method can be a valuable tool in a material scientist’s testing arsenal.

Unfortunately, reported academic and industrial applications using the Iosipescu test are limited [11] and of existing research, the overwhelming majority is focused on Iosipescu laminates. Deng et al. [20] remains one of the only cases that has explored the Iosipescu tow test as an effective means to observe interface interactions. To ensure this technique of investigating composite bonding remains relevant and applicable, further research into unknown testing variables such as tow size, resin permeability and fiber chemistry is required.

This paper addresses major concerns relating to the Iosipescu tow test; namely the influence of tow bundle size, influences of tow wetting, effects of altered interface chemistry on testing validity and the correlation between SFFT results.

## 2. Materials and Methods

### 2.1. Raw Materials

Three types of carbon fiber were used within this study; standard T300 (Toray, Tokyo, Japan) and two surface modified variants of the same T300 fiber mat. These variants will be referred to for the remained of this study as “T300-Amine” and “T300-Ester”. The procedure of modifying standard T300 into to T300-Amine and T300-Ester fibers through a process of desizing and electro-grafting is detailed in Section 2.1.1. A 1 m^2^, 3 k tow plain weave fabric was purchased with individual tows extracted from this weave. Fabrication and sizing specifications of fiber are ambiguously marked in accordance with Toray classification, however for further information refer to the materials data specifications [21].

Single fiber tensile testing was conducted on each fiber type to determine mechanical properties. 75 singular fibers of each three variants were carefully removed from respective tow bundles and cut to a length of approximately 50 mm. A 0.8 g pre-weight was attached to one end of the fiber and then loaded into a Favimat single fiber testing robot (https://www.textechno.com/product/favimat-with-airobot2-and-autofeed/) which determines the tensile strength, elongation at break, tensile modulus, and fiber diameter, normalized by linear density found via vibroscope. Results of tensile strength, elastic modulus and maximum elongation are presented in Table 1.

Results indicate that T300-Amine fibers and T300-Ester fibers both have a statistically significant reduction to elastic modulus. This reduction is curious as the elongation percentage remains comparable for all three fiber types while the break tension to failure slightly drops. As Youngs modulus is defined as the ratio of stress divided by elongation; this data suggests that the ability to carry stress is slightly decreased for the functionalized fibers or they are more susceptible to elongation. This phenomenon is postulated to be an artifact of the desizing, washing and electro grafting processes which may all introduce fiber surface flaws. This was experimentally observed by the decreased break tension between amine fibers and ester fibers. As the amine functionalized fibers required additional electrolysis and washing to reduce the nitro moieties to an amine, they experienced even greater reductions than ester fibers to both modulus and ultimate break tension. As such, the introduction of surface flaws due to handling and desizing are being attributed to decreased modulus. Despite these differences, error margins were observed to overlap for all fiber types. While these changes in mechanical performance are noted, results do not provide any indication of the quality of interfacial bonding between the fibers and resin matrix. Hence this investigation remains justified.

**Figure 2:**
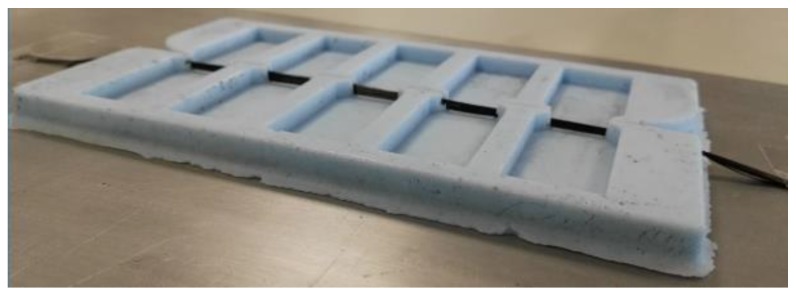
Fibers tensioned across silicon Iosipescu mould prior to resin injection.

Resin used within this study was epoxy RIMR 935 mixed with RIMH 937 hardening agent at a part by weight ratio of 1:0.38 respectively (Hexion, Columbus, OH, USA). Once combined, the resin was thoroughly mixed for 20 min then placed under 100 kPa vacuum for degassing. 3 mL syringes were used to inject the resin into a specially designed Iosipescu (Figure 2) and SFFT moulds (see Figures S1 and S2, Electronic Supplementary Information (ESI)). Resins were allowed to cure at room temperature for 48 h then post-cured at 100 °C for 12 h. Although not a measure of interfacial shear strength, Iosipescu samples of neat resin were created and observed to fail at an average break load of 2560 N with a standard deviation of 255 N.

#### 2.1.1. Electro-Grafting Procedure

Carbon fiber electrodes were prepared using approximately 20 × 20 cm^2^ of a carbon fiber fabric (2 × 2 twill weave, desized by refluxing acetone for 24 h). Copper adhesive tape was applied to one edge of the carbon fiber mat to ensure adequate connection between the fibers and the electrode clamp, this mat served as the working electrode. Electrochemical measurements were conducted using a Metrohm PGSTAT potentiostat (Herisau, Switzerland); utilizing a three-electrode setup with a LF-2 leak free electrode (filling electrolyte 3 M KCl) half-cell as the reference and a platinum mesh wire as the counter. Before scanning, the system was purged using N_2_ gas to remove any dissolved oxygen and the cyclic voltammetric scans were performed utilizing a 2 V potential window between +1.0 V and −1.0 V, at a scan rate of 10 mV/s for 6 scans. The solution was then agitated to disrupt the formation of diffusion layers and the sweeps repeated.

The electrolyte solution consisted of 1 mmol of the select diazonium salt (Nitrobenzene diazonium tetrafluoroborate for the amine, benzocaine diazonium tetrafluoroborate for the ester) in 250 mL acetonitrile. Tetrabutylammonium hexafluorophosphate (TBAPF_6_) to a concentration of 0.1 M was also added to serve as a supporting electrolyte. After functionalization, the fibers were then rinsed thoroughly with a series of organic solvents (chloroform, dichloromethane, ethanol and acetone), followed by drying under reduced pressure for 24 h to ensure the removal of residual solvent. The T300-Amine mat was then subjected to further electrolysis to reduce the nitro moiety to an amine. This was achieved in a 0.1 M concentration of KCl in a 9:1 mixture of ethanol and water, performing a cyclic voltammetric scan with a potential window of −1.5 V to +1.0 V and a scan rate of 10 mV/s for 6 scans, before the solution agitated and the fibers removed. Finally, the fibers with a series of organic solvents (chloroform, dichloromethane, ethanol and acetone) were allowed to dry under reduced pressure for 24 h.

Synthesis of aryldiazonium salts were carried out according to our previously reported syntheses, along with further discussion on the electrochemistry involved [22].

### 2.2. Iosipescu Testing

**Figure 3:**
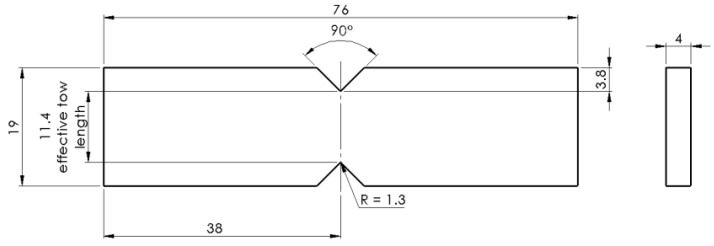
Iosipescu specimen geometry (measurements in mm).

Iosipescu test specimens were manufactured by tensioning tow bundles of fibers across a specially designed V-notch silicon mould (Figure 2 and Figure 3). An aluminum male mould was manufactured using a CNC router (see ESI) and Silastomer P40 (Dalchem, Cheltenham, Australia) poured into the male mould. Silicon was cured at room temperature for 24 h before being removed.

Fibers were subsequently tensioned across the center cavity of the silicon mould and sticky tape used to maintain tension. Resin was then injected into the mould using a 3 mL syringe until resin depth reached 4 mm. Samples cured as specified prior (Section 2.1). Once cured, samples were removed from the silicon mould and a Dremel 3000 rotary cutter (Bosch, WI, USA) used remove excess fiber and trim the V-notch shape in accordance with ASTM D5379 [23]. Edges were also briefly polished using a 500 grit polishing pad (Flexovit, Saint-Gorbain, France).

**Figure 4:**
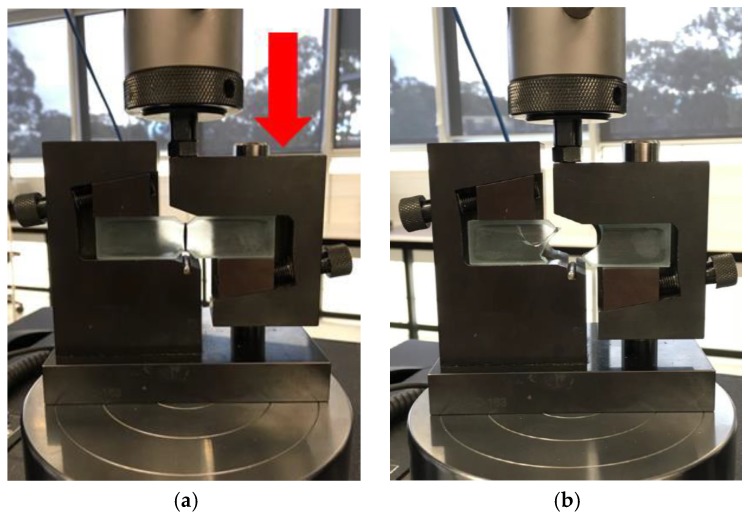
Specimen prepared for loading (**a**) Post fractured specimen (**b**).

Specimens were then loaded into a specially designed test fixture known as the modified Wyoming fixture (Wyoming Test Fixtures Inc., Salt Lake City, UT, USA, Figure 4). The specimens were loaded within the fixture, which was attached to a 10 kN load cell within a 5966 Instron universal testing machine (Instron, Norwood, MA, USA). Specimens were aligned using a marking rod and clamps either side of the specimen center tightened to secure samples into place. Testing is conducted by one side of the test rig remaining static while the other end is moved downward via displacement control on the vertical axis. Rate of vertical compression was 2 mm/min, creating a region of pure shear across the specimen’s center [13,14,15,17]. Dependent on interfacial bonding between the fibers and resin, the performance varies accordingly. IFSS was calculated using Equation (1) where *P* is the load at failure and *A* is the cross-sectional area of thickness multiplied by effective tow length.

(1) $$\;\tau =\frac{P}{A}\;$$

For Section 3.1 (tow sensitivity study), tow bundle sizes of 3 k, 6 k, 9 k, 12 k, 15 k, 18 k and 24 k where tested where “k” refers to the number of thousand fiber filaments in a tow in accordance to industry vernacular [24]. Variation in tow size was obtained by removing individual 3 k tows from the T300 mat, and then tensioning across the Iosipescu mould one at a time to obtain the desired filament quantity. Thereby all tow sizes were created by combining multiple 3 k tows together (e.g., 12 k = 4 × 3 k tows).

For Section 3.3 (interface chemistry sensitivities), tow size was held consistent at 9 k. This was done to ensure sufficient fiber wetting and to allow the dominance of the fibre interface. Fracture patterns observed during testing are discussed in Section 3.2 (fracture pattern analysis, see supplementary information Figure S3) with relation to recorded performance and resin permeability.

### 2.3. Single Fiber Fragment Testing (SFFT)

SFFT provides the ideal wetting conditions of microscale single fiber interaction between the three fiber types and the resin matrix. Single fiber fragmentation samples were created by isolating single fibers from tow bundles and tensioning them across a specially designed silicon mould (see ESI). Fibers were pre-tensioned at 3.4 mN on either end to ensure straightness and resin carefully injected using a 3 mL syringe. Subsequent to the curing process, samples were demoulded and tensioned in the universal testing machine at an elongation rate of 0.05 mm/min for 50 min.

An Olympus DP70 polarized optical microscope (Shinjuku, Tokyo, Japan) was then used to count fragment lengths (*l*) and IFSS determined using Equation (2) (where $l_{c}$ is critical crack length (Equation (3)), d is fiber diameter and $\sigma_{f}$ is ultimate fiber tensile strength). Values d and $\sigma_{f}$ were determined through the aforementioned Favimat Robot testing and a Weibull probability analysis. Weibull analysis of Favimat data also provides the Weibull shape parameter (m) and the probability distribution (P).

(2) $$\;\tau =\frac{\sigma_{f}d}{2l_{c}}\;$$

(3) $$\;l_{c}=\frac{4}{3}\;\bar{l},\;$$

SFFT provides the ideal wetting conditions of microscale single fiber interaction between the three fiber types and the resin matrix.

## 3. Results

### 3.1. Fiber Tow Sensitivity of the V-Notch Shear Test

**Figure 5:**
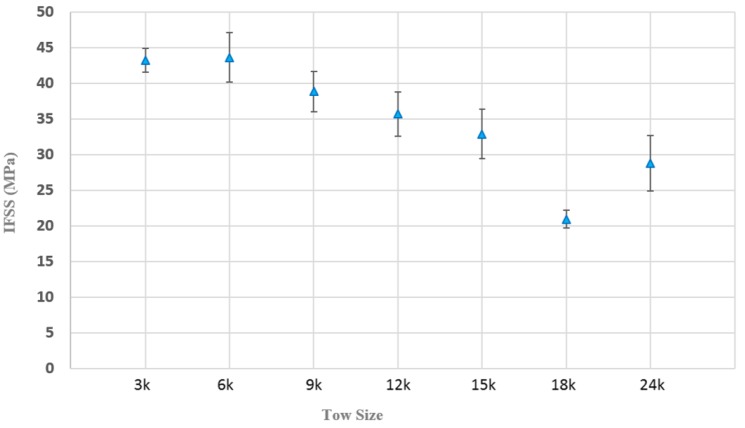
IFSS results of 3 k, 6 k, 9 k, 12 k, 15 k, 18 k and 24 k T300 fibers.

The IFSS presented in Figure 5 provides values of the seven different T300 tow bundle sizes investigated. Results showed the 3 k and 6 k tows to have the greatest IFSS values, which were 11.6% and 12.7% greater than neat resin coupons. Interestingly the 9 k tow bundle had a similar failure load to that of the neat resin. This raises a question as to the influence of tow embedding. Is the 9 k tow bundle having any mechanical effect on the shear stress plane or is stress distribution completely resin dominated? To explore this question, 9 k tow bundles were used as the control in Section 3.3 (interface interaction sensitivities). Later results will show that indeed the 9 k tow interactions were playing a role on performance during this phase of testing.

**Figure 6:**
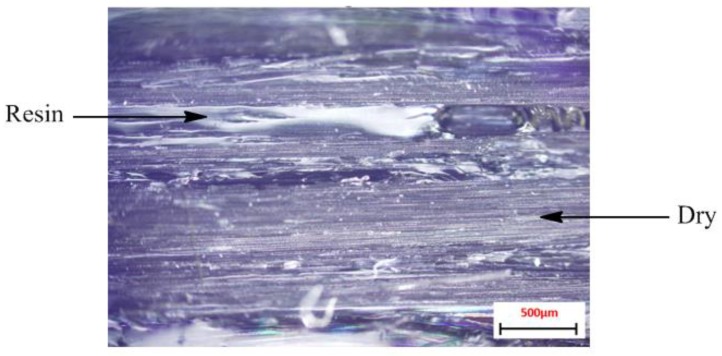
Optical microscope image of dry fibers for sample with central tow splitting.

As tow size increased from 9 k through to 12 k and 15 k, gradual decreases in IFSS were observed. 12 k and 15 k IFSS values were 35.70 ± 6.2 MPa and 32.85 ± 6.94 MPa, respectively (Table 2). This decrease in performance may be attributed to poor fiber wettability manifesting as localized void regions within the tow due the difficulty of the epoxy resin to completely penetrate these thicker tows. This is undesired, as these dry locations within the tow will cause premature failure. This is likely an artefact of atmospheric curing conditions, which is not representative of a resin transfer infusion (RTI) where a high pressure is applied to increase permeation of the resin through the fibers. An example of the poor wetting out of these fibers is shown in Figure 6, demonstrating the dry fibers that have initiated failure of the specimen.

Post analysis of fracture sites using an optical microscope supports this hypothesis, however, the greatest indication of poor fiber wettability was observed when tow size was further increased to 18 k and 24 k. At these tow sizes, IFSS values dropped to 20.92 ± 2.47 MPa and 24.40 ± 2.40 MPa, respectively (Table 2).

**Figure 7:**
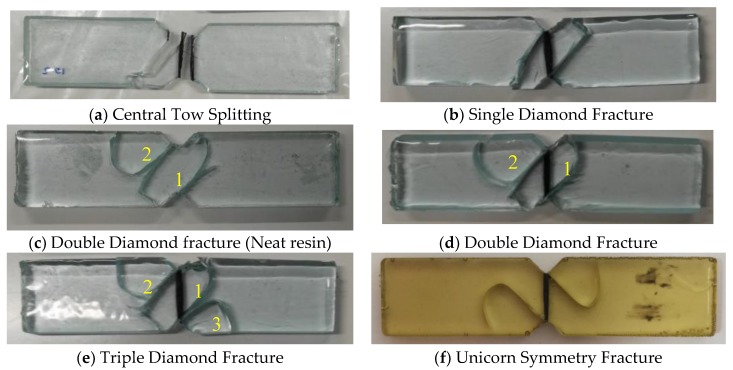
Catalogue of commonly observed fracture patterns during Iosipescu tow testing (**a**–**f**).

For both the 18 k and 24 k tow specimens, two out of every five specimen samples failed under central tow splitting (Figure 7a). While this failure mode is an acceptable failure state under Iosipescu protocols (ASTM D5379), in the context of this study it is considered to be a premature failure. This is due the fact that the failure does not occur at the interface, rather through the tow because of poor wetting. As the failure is not related to shear forces at the interface, the data obtained from these samples were not included in the average IFSS values. However, their prominence as a failure mode with increased tow size coupled with significantly decreased IFSS values suggests that there is undoubtedly an ideal tow size required when using the Iosipescu tow test. Without considering tow bundle size effects, results may provide inaccurate indicators of IFSS especially where resin permeability becomes a concern. Researchers are encouraged to conduct their own sensitivity studies where applicable and exercise caution when testing tows greater than 12 k.

In summary, 3 k, 6 k and 9 k tow bundles were observed to be the most suitable tow sizes for the reliable determination of IFSS via this method. However, as tow size increased from 9 k, to 12 k and 15 k bundles, IFSS was observed to slowly decrease, and thus more caution is advised when using tows of this size. At 18 k and 24 k tow bundles this issue of poor fiber wetting became self-evident due to the introduction of central tow splitting which also coincided with decreased IFSS. It is recommended at these tow sizes that the fibers be infused with resin under pressure or a very large volume of samples obtained to minimize effects from premature failure.

### 3.2. Fracture Pattern Analysis

As testing using a single tow Iosipescu test is still under-developed, a catalogue of commonly occurring fracture patterns (such as those observed within this study) may be highly beneficial for others looking to employ this methodology. Figure 7a–f demonstrates a grouping of common failures observed during testing.

- Central Tow Splitting: This mode of failure is characterized by a very low fracture load and poor interfacial adhesion. While it is an acceptable mode of failure in accordance with ASTM D5379, researchers are encouraged to conduct post analysis of fracture sites to conclude failure was indeed related to interface delamination and not poor fiber wetting which is a more likely cause. (Failure load—Low).
- Single Diamond Fracture: This is the most commonly observed fracture pattern in single tow Iosipescu testing. It is characterized by a 45 degree “diamond” breakage that runs parallel to the V-notch walls and may fracture fibers close to the notch root. (Failure load—moderate).
- Double diamond fracture (neat resin): All neat resin samples were observed to fracture identically in this fracture pattern. Aesthetically comparable to the “single diamond fracture” shape, however also ejects a secondary section (2) off the coupon upon fracture. (Failure load—moderate to high).
- Double diamond fracture (tow): The same fracture pattern as observed by the neat resin specimens, though with a tow embedded. Failure load was observed to always be larger than that of the single diamond fracture pattern. (Failure load—moderate to high).
- Triple diamond fracture: A further variant of the diamond fracture pattern however characterized by the breakage of three distinct sections; the central diamond (1) and two adjacent nubs (2 and 3). This mode of failure is observed to provide the best interfacial properties. (Failure load—extremely high).
- Unicorn Symmetry Fracture: While not a part of this study, a theoretically perfect fracture was observed during in-house testing. Representative of the von-mises stress contour plots, this fracture was an extremely unique occurrence presenting an experimentally ideal failure with respect to theoretical stress mapping. (Failure load—moderate).

### 3.3. Interface Chemistry Sensitivity

**Figure 8:**
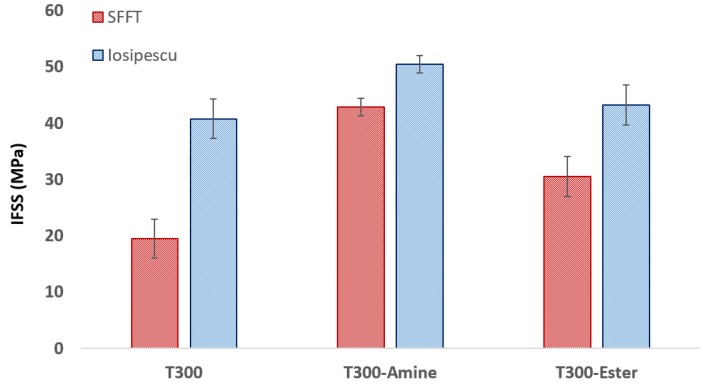
SFFT and Iosipescu IFSS results of T300, T300-Amine and T300-Ester fibers.

To observe the effects of interface chemistry, three fibers were tested under both SFFT and Iosipescu tow conditions. Each fiber set had a different surface chemistry installed using reductive electrografting of aryldiazonium salts, as reported elsewhere [25,26,27]. The IFSS results (from SFFT) of the three fiber configurations are presented in Figure 8. Commercial T300 fibers were observed to have the lowest IFSS, at 19.46 ± 0.5 MPa. The poor adhesion also suggests that the compatibility of the T300 epoxy sizing is not optimal for interfacial adhesion in this resin system. Conversely, T300-Amine and T300-Ester fibers, both of which were desized, possessed larger IFSS (42.85 ± 6.1 MPa, and 30.51 ± 5.6 MPa, respectively). While the interfacial adhesion gains from the T300-amine fibers are most likely attributable to covalent cross-linking with the epoxy resin, the ethyl ester moiety is unable to participate in similar interactions. Based on our previous work [25,26,27], we have shown that simply the presence of a small molecule on the fiber is enough to induce improvements in IFSS due to a type of ‘molecular drag’ which is occurring through the polymer phase. Interestingly, the standard deviation of the T300 fibers was significantly smaller than the amine and ester functionalized variants. This suggests that sizing acts to protect fibers from damage and creates a more chemically homogenous surface which reduces variation in experimental results. This may also relate to the homogeneity of the surface modification process being inconsistent along the length of the fiber as reactivity of the graphite crystals (edge versus basal plane) will vary according to precursor, processing conditions, and surface treatment during manufacture.

One of the aims of this investigation was to observe the effects of interface chemistry variables on the Iosipescu tow test. Figure 8 (red) provides the microscale IFSS foundation that the mesoscale Iosipescu test can be compared against. These SFFT results are interesting in their own right and show surface chemistry alone to be a dominating factor for improved adhesion. Figure 8 provides the scale comparison of SFFT and Iosipescu tow test IFSS results using a 9 k tow bundle, as this was determined to be optimal (Section 3.1, above).

For all configurations, the IFSS values using the Iosipescu test (IOS in Figure 8) were found to be substantially greater than the corresponding SFFT values. Standard T300 fibers were found to have the largest disparity between methods at 21.4 MPa (Table 3), followed by T300-Ester fibers (12.7 MPa) and T300-Amine fibers (7.6 MPa). While these differences show a disconnect between micro- and meso-scale values, the trends observed across the two testing scales are comparable. The T300-Ester and T300 samples were statistically indistinguishable (*p* = 0.55), when analyzing the IOS test while at the single filament level the difference in IFSS is clearly visible (*p* < 0.05). This suggests that while the trends are generally similar, the fidelity of discriminating between similar IFSS values is reduced (Table 3). Therefore, the Iosipescu tow level test may be successfully used to compare interfacial adhesion for modified carbon fibers, but may only distinguish between significantly different IFSS values. While this may be seen as a limitation, it is important that this analysis be used in conjunction with other interfacial adhesion studies, such as the SFFT. Indeed, using both of these tests in conjunction will provide differing perspectives on what factors may govern the design of optimal interfacial adhesion in composite materials.

Interestingly, while only limited publications investigating correlations between SFFT and Iosipescu testing exist, this phenomenon of IFSS discrepancy between testing scales has been observed and documented prior. In a study conducted by Herrera-Franco et al. [1] Iosipescu values were found to be between 36.7% larger than SFFT values for AU-4 and AS-4 fibers in an 828 epoxy matrix. Similarly, Drzal et al. [28] documented Iosipescu IFSS values to be greater than those of SFFT, however, they observed the strength of an interface to have a direct correlation to how pronounced differences may be. By broadly quantifying three interfaces as “weak”, “moderate” and “strong”, the study found differences between IFSS to be 47.5%, 39.9% and 15.2%, respectively. Hence, as interface strength increases, the IFSS disparity across testing platforms decreases. This same trend was observed in Figure 8, which showed the IFSS values for both tests to be most comparable for the T300-Amine fibers.

There are many reasons that may be causing the disparity between SFFT and Iosipescu tow test results. It could be that due to the inability of SFFT to account for shrinkage pressure and Poission contractions, IFSS of a single fiber microscale test provides underestimations of IFSS [29]. Conversely the creation of localized regions of plasticity may be occurring nearing failure thereby artificially the calculated IFSS values of the Iosipescu results. This would be in line with some modelling research conducted on laminates [12]. Regardless, the disparity does not detract from the same conclusions being drawn by the two tests and provided that both are conducted carefully, either one can be a valuable tool in acquiring information regarding interfacial adhesion. This is the same dilemma that has existed for countless years between SBS testing and SFFT; however both are still used as quantifiers of interfacial performance while providing different IFSS/ILSS values [1,28,30]. The Iosipescu test too should be considered valuable on its own merits.

When considering interfacial adhesion as a function of fiber treatment conditions, undoubtedly the effects of electro-grafting amine and ester groups are prominent and observable at a microscale. Though some resolution is lost when investigated at the mesoscale, variance is still significant.

## 4. Conclusions

The Iosipescu tow test can be used to determine IFSS for fiber tows in a resin matrix. However, as tow sizing increases resin wettability may become a concern. It is recommended that if the tow size used exceeds 12 k, a tow sensitivity study and/or post analysis of fracture sites should be conducted. This ensures resin permeability is not a factor during failure. Similarly, if failure occurs via “central tow splitting”, the fracture site should be analyzed to ensure that failure was indeed at the interface and not a result of poor fiber wetting. A comparison of different fiber surface chemistries also revealed that the SFFT and Iosipescu test may yield the same performance trends, however, the Iosipescu tests provides a reduced resolution between two methods. Also, values from Iosipescu testing are usually larger than those from SFFT for reason explored in this report.

Authors would note that no experimental method for interface testing is perfect. Microscale tests such as the SFFT and macroscale tests such as the SBS test all have inherent shortcomings. The Iosipescu test is one of the few methods to provide a pure region of shear stress in which an interface can be tested and has the versatility to be used for mesoscale “tow” testing thereby allowing investigation of specifically neighboring fiber interactions. While the Iosipescu test can provide a quick and relatively accurate determination of IFSS, it should not be used in isolation (nor should any other IFSS test). Especially in its current state of limited academic exposure, the test is an invaluable asset in providing complimentary information to other tests such as SBS and SFFT.

The Iosipescu tow test was found to be a valid means of determining IFSS and is comparable to SFFT, albeit, better performing with high IFSS fibers. An additional catalogue of potential failure modes experienced during testing was also provided to assist future researchers in conducting Iosipescu tow testing.

## Figures and Tables

**Table 1:** Carbon fiber material properties table.

Property	T300	T300-Amine	T300-Ester
Elongation (%)	1.7 [0.3]	1.7 [0.3]	1.7 [0.2]
Break Tension (GPa)	3.7 [0.6]	3.2 [0.6]	3.5 [0.6]
Modulus (GPa)	239.4 [16.2]	206.4 [7.4]	227.8 [6.6]

[] Standard Deviation.

**Table 2:** IFSS results of seven varied tow bundle sizes tested under Iosipescu testing.

Specification	3 k	6 k	# 9 k	12 k	15 k	18 k	24 k
IFSS	43.21	43.61	38.81	35.70 *	32.85 *	20.92 *	24.40 *
(SD)	(3.37)	(6.93)	(5.70)	(6.20)	(6.94)	(2.47)	(2.40)
PF	0	0	0	1	0	2	2

PF = Premature failures; SD = Standard deviation * Denotes statistical significance (*p* < 0.05) with respect to 3 k specimen; # 9 k tow size data result is also used for testing comparison in Section 3.3.

**Table 3:** Comparison of testing method on fiber types.

Specification	T300		T300-Amine		T300-Ester	
	SFFT	IOS	SFFT	IOS	SFFT	IOS
IFSS (MPa)	19.46	40.80	42.85	50.45	30.51	43.26
St. Dev	0.48	1.55	6.1	3.55	2.5	3.46
Increase (MPa) ^a^	-	21.4	-	7.6	-	12.7

**^a^** Increase relative to IFSS determined by SFFT. IOS—Iosipescu Test

## Supplementary Materials

The following are available online at http://www.mdpi.com/1996-1944/11/9/1786/s1, Figure S1: Aluminium male section for Iosipescu moulds, Figure S2: Silicon SFFT mould with clear resin and fibers tensioned across the center, Figure S3. Example of ongoing testing of epoxy resin and tow sizes with other resin systems. (2,2-Bis[4-(glycidyloxy)phenyl]propane/4,4′-Methylenedianiline) DGEBA/DDM.
