# Supplementary material for: Effect of Tow Size and Interface Interaction on Interfacial Shear Strength Determined by Iosipescu (V-Notch) Testing in Epoxy Resin
Source: Materials (Basel). 2018 Sep 19;11(9):1786. doi: 10.3390/ma11091786 (PMC6163553; doi:10.3390/ma11091786)
Supplement: Supplementary file 1 [file materials-11-01786-s001.pdf]

Supporting Information for:

Effect of tow size and interface interaction on interfacial shear strength determined by Iosipescu (V-Notch) testing.

Filip Stojceveski * Andeas J. Hendlmeier, James D. Randall, Chantelle L. Arnold, Melissa K. Stanfield, Daniel J. Eyckens, Richard Alexander and Luke C. Henderson

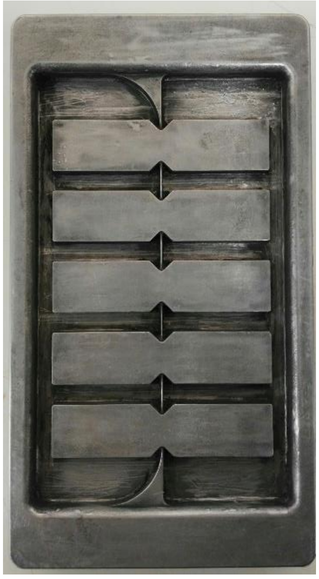

Figure S1 (ESI). Aluminium male section for Iosipescu moulds.

The CAD model of the male mould was drawn with SolidWorks 2017 (Dassault Systèmes, Vélizy-Villacoublay, France).

The cutting path (g-code) was generated using Siemens NX10.0 (Siemens PLM, Plano, TX, United States).

The mould was CNC machined from 7075 Aluminium (Bohler, Uddeholm, Dandenong South, Victoria, Australia) on a Datron M7 HP (Datron AG, Mühltal, Germany) using Datron cutting tools (Datron AG, Mühltal, Germany).

After machining the male mould was polished with white polishing compound (Josco, Dandenong, Victoria, Australia) on a 100 mm white stich wheel (Polish Up, Woombye, Queensland, Australia) followed by blue finishing polishing compound (Polish Up, Woombye, Queensland, Australia) on a 100 mm swansdown wheel (Polish Up, Woombye, Queensland, Australia), finally, the part was degreased with whiting powder (Polish Up, Woombye, Queensland, Australia) before use. The final products is provided in Figure S1.

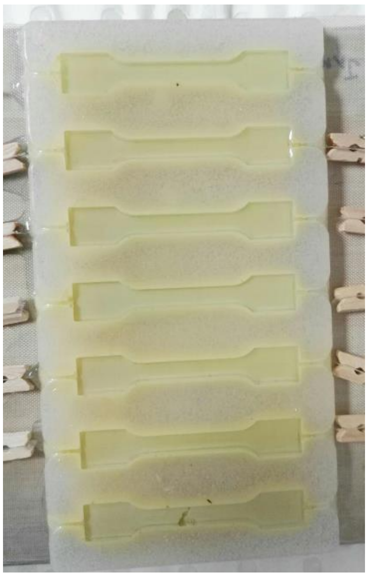

Figure S2 (**E**SI). Silicon SFFT mould with clear resin and fibers tensioned across the center.

Figure S2 provides an image of the silicon mould required for the fabrication of single fiber fragmentation specimens. These are manufactured according to manufacturers specifications. V-notch shear specimens, after evaluation have been provided in Figure S3. These show the variations in fracture type as discussed within the main text of the manuscript.

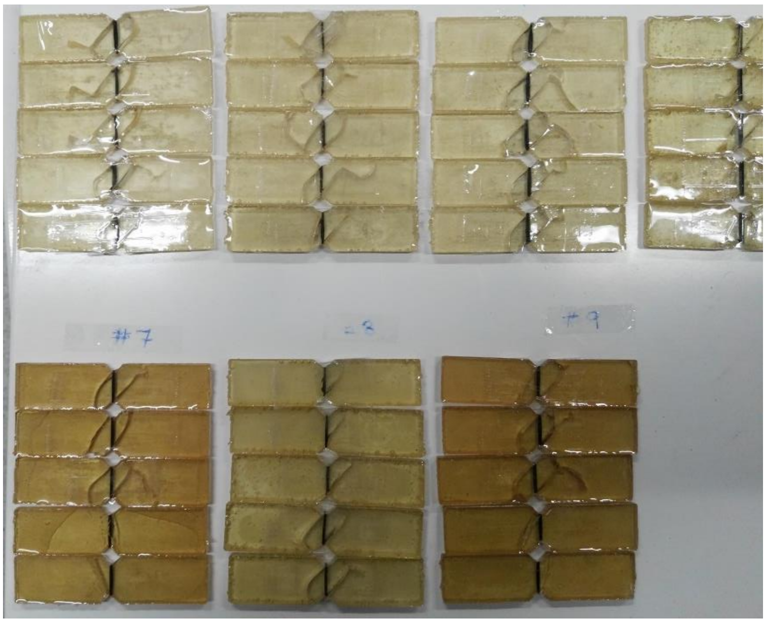

Figure S3. Example of ongoing testing of epoxy resin and tow sizes with other resin systems. (2,2-Bis[4-(glycidyloxy)phenyl]propane/4,4′-Methylenedianiline) DGEBA/DDM.
